# A New Zn(II) Metal Hybrid Material of 5-Nitrobenzimidazolium Organic Cation (C_7_H_6_N_3_O_2_)_2_[ZnCl_4_]: Elaboration, Structure, Hirshfeld Surface, Spectroscopic, Molecular Docking Analysis, Electric and Dielectric Properties

**DOI:** 10.3390/ma15227973

**Published:** 2022-11-11

**Authors:** Chaima Ayari, Abdullah A. Alotaibi, Mohammed A. Baashen, Fouzia Perveen, Abdulhadi H. Almarri, Khalid M. Alotaibi, Mohammed S. M. Abdelbaky, Santiago Garcia-Granda, Abdelhak Othmani, Cherif Ben Nasr, Mohamed Habib Mrad

**Affiliations:** 1 Materials Chemistry Laboratory, Faculty of Sciences of Bizerte, University of Carthage, Zarzouna, Bizerte 7021, Tunisia; 2 Department of Chemistry, College of Sciences and Humanities, Shaqra University, Ad-Dawadmi 11911, Saudi Arabia; 3 School of Interdisciplinary Engineering and Sciences (SINES), NUST, H-12, Islamabad 44000, Pakistan; 4 Department of Chemistry, University College of Al-Wajah, University of Tabuk, Tabuk 71421, Saudi Arabia; 5 Department of Chemistry, College of Science, King Saud University, Riyadh 12271, Saudi Arabia; 6 Department of Physical and Analytical Chemistry, University of Oviedo-CINN, 33006 Oviedo, Spain; 7 Laboratory of Material Physics: Structures and Properties, LR01 ES15, Faculty of Sciences, University of Carthage, Zarzouna, Bizerte 7021, Tunisia

**Keywords:** Zinc (II) complex, hydrogen bonds, AC conductivity, FT-IR, molecular docking study

## Abstract

The slow solvent evaporation approach was used to create a single crystal of (C_7_H_6_N_3_O_2_)_2_[ZnCl_4_] at room temperature. Our compound has been investigated by single-crystal XRD which declares that the complex crystallizes in the monoclinic crystallographic system with the *P*2_1_/*c* as a space group. The molecular arrangement of the compound can be described by slightly distorted tetrahedral ZnCl_4_^2−^ anionic entities and 5-nitrobenzimidazolium as cations, linked together by different non-covalent interaction types (H-bonds, Cl…Cl, π…π and C–H…π). Hirshfeld’s surface study allows us to identify that the dominant contacts in the crystal building are H…Cl/Cl…H contacts (37.3%). FT-IR method was used to identify the different groups in (C_7_H_6_N_3_O_2_)_2_[ZnCl_4_]. Furthermore, impedance spectroscopy analysis in 393 ≤ T ≤ 438 K shows that the temperature dependence of DC conductivity follows Arrhenius’ law. The frequency–temperature dependence of AC conductivity for the studied sample shows one region (E_a_ = 2.75 eV). In order to determine modes of interactions of compound with double stranded DNA, molecular docking simulations were performed at molecular level.

## 1. Introduction

Zinc (belonging to the fourth period of T.P) is one of the most essential metallic entities in the human body and has a major role in biological systems [1]. Zn has a bacteriostatic behavior on many microorganisms [2,3], and has various industrial applications such as in food, pharmacology, power leading, materials, and chemistry [4,5,6,7]. Furthermore, Zn (II) is often stabilized by a tetrahedral coordination environment to fill out the 4s and 4p orbitals [8,9,10]. The complexes based on Zn have a high-performance property focusing on photoluminescence, letting them to be adapted as light sensors, biological imaging probes, and electrochemical machines [11,12,13,14,15,16,17,18,19,20,21]. The interaction types X–H…A which are classified as non-covalent, metallophilic, halogen–halogen, X–H…π, π…π, lead to mixing two entities (organic and inorganic parts) together in a single hybrid derivative, resulting in structure stability [22,23,24,25,26,27,28,29,30,31,32,33]. 

In organic entities that include heteroatoms in their structures, π-electrons come from the aromatic ones, and conjugated bonds can be adapted in various fields, as mentioned by several researchers in their work [34,35,36]. Benzimidazole derivatives have an important role in the domain of chemistry especially medicinal chemistry. The benzimidazole derivatives are used as fungicides in agriculture due to the high activity against pathogenic and non-pathogenic fungi [37,38]. Recent research have demonstrated that benzimidazole derivatives exhibit very good steel-inhibiting performance in acid solution [39,40,41,42,43,44].

In this article, we present an in-depth study of a new material synthesized by an acid–base reaction between zinc chloride and the ligand 5-nitrobenzimidazole, by using SCXRD, PXRD, FT-IR spectroscopy, molecular docking study and impedance spectroscopy.

## 2. Experimental Part

### 2.1. Chemical Preparation

By the acid–base reaction between methanolic solution containing 5-nitrobenzimidazole (0.3262 g, 2 mmol, purity 98%, Sigma-Aldrich, Burlington, MA, USA) and solution containing ZnCl_2_ (0.2445 g, 1 mmol, purity ≥98%, Sigma-Aldrich) dissolved in 10 mL of hydrochloric acid (1M, purity 36–38%, Sigma-Aldrich), we produced crystals of (C_7_H_6_N_3_O_2_)_2_[ZnCl_4_] by evaporation method at T = 24 °C. Colorless prism crystals appeared after three weeks (Yield: 78%).

**Scheme 1:**
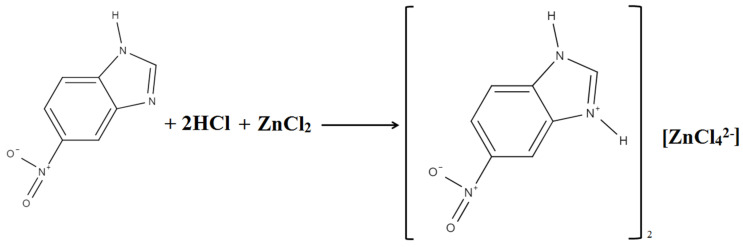
Synthesis of (C_7_H_6_N_3_O_2_)_2_[ZnCl_4_].

The reaction Scheme 1: 

The CHN-elemental analysis declares: C: 31.46%; 31.39%/N: 15.70; 15.65/H: 2.26%; 2.18%/O: 11.95%; 11.88%.

### 2.2. Methods Details 

Using Xcalibur, Ruby, Gemini diffractometer equipped with MoK_α_ radiation (0.71073 Å) at 293 K, we succeeded in identifying the crystallographic data of (C_7_H_6_N_3_O_2_)_2_[ZnCl_4_]. The refinement was performed by the SHELXL program version 2018/1 [45]. By exploiting the Diamond program, we succeeded in drawing the graphs explaining the structure of the material [46]. The ORTEP was drawn by the Mercury software [47]. All crystallographic details are displayed in Table S1. To acquire the infrared spectrum of (C_7_H_6_N_3_O_2_)_2_[ZnCl_4_], a spectrophotometer called Nicolet Impact 410 FT-IR was used according to the manufacturer’s instruction (SpectraLab Scientific Inc., Markham, ON, Canada). The measurement of the real Z’ and imaginary Z” impedance characteristics was carried out for 393 ≤ T ≤ 438 K and for 10^1^ ≤ f ≤ 10^7^ Hz by using the Hewlett Packard 4192A analyzer and with a disc of pellets approximately 6 mm in diameter and 1.2 mm in thickness.

Chemical computing Inc., Molecular operating environment MOE 2017 program was applied for molecular docking simulations of interaction analysis of compounds. Crystal structure of (C_7_H_6_N_3_O_2_)_2_[ZnCl_4_] was imported to MOE interphase for optimization of the structure through the MOPAC 7.0 level of theory. The model was fetched from the database after geometry fixing. X-ray crystallographic structure of DNA leading to PDB ID: 6TNY and resolution of 3.0 Å was uploaded from the Protein Data Bank [48]. 6TNY was protonated and optimized through the protonate-3D menu after the removal of water molecules from the 6TNY structure. In order to investigate docking analysis, coordinates of 6TNY were optimized using AMBER force field and semi-empirical PM3 approaches. Relaxed coordinates possessed the lowest energy and stable conformation for the uploaded functional function calculations. (C_7_H_6_N_3_O_2_)_2_[ZnCl_4_] compound optimized geometries were subjected to methodical molecular docking taking 6TNY as receptor at default parameters with an RMS gradient in the order of 0.01 kcal/mol. Dummy atoms were generated for finding interactions sites of 6TNY using Site Finder. Many docking essays were carried out to performed runs to obtain the final docking poses as perfect as possible. The interaction energy of (C_7_H_6_N_3_O_2_)_2_[ZnCl_4_] compound with 6TNY was determined at each step of the simulation. The rest of the parameters were kept as default [49]. 

## 3. Discussion Part

### 3.1. Crystal Structure Details

**Figure 1:**
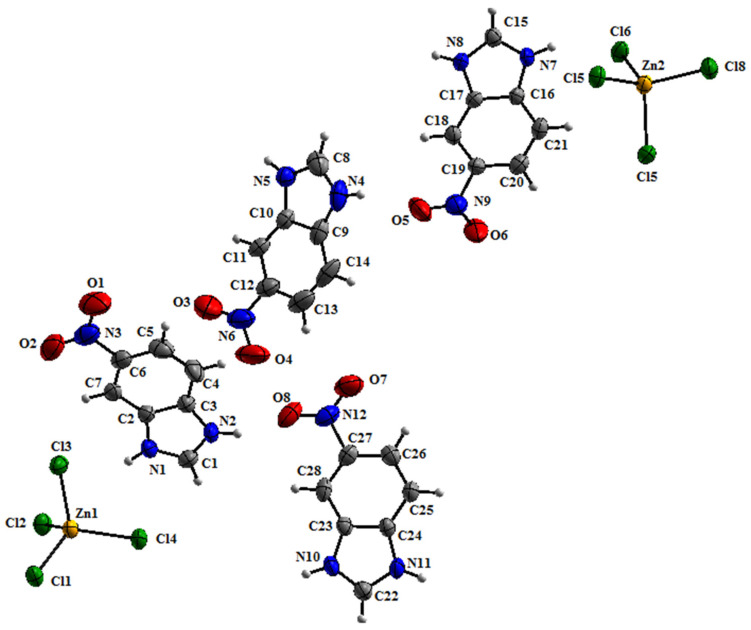
Crystal unit lattice of (C_7_H_6_N_3_O_2_)_2_[ZnCl_4_].

From the results obtained by SCXRD technique, the ORTEP of the bis(5-benzimidazolium) tetrachlorozincate (II) compound is constituted by two independent [ZnCl_4_]^2^-anions and four independent 5-nitrobenzimidazolium cations (Figure 1).

**Figure 2:**
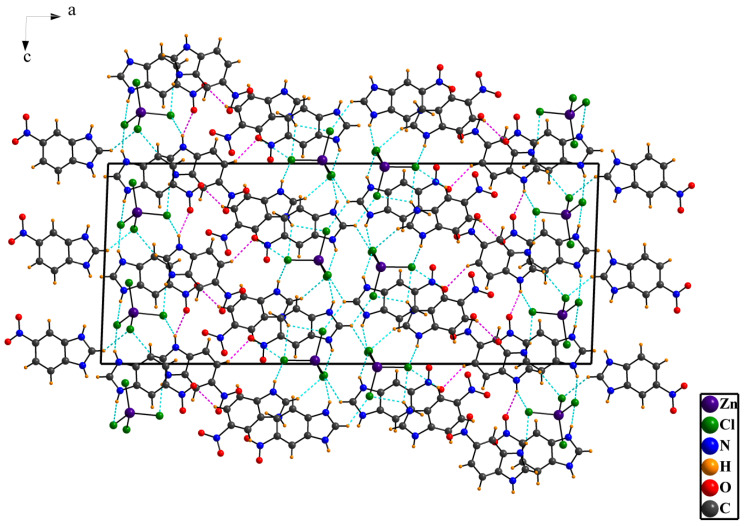
Projection in the plane (a,c) of the atomic arrangement of (C_7_H_6_N_3_O_2_)_2_[ZnCl_4_]. The red and cyan lines correspond to the H-bonds.

The solid-state arrangement of (C_7_H_6_N_3_O_2_)_2_[ZnCl_4_] can be described as an alternation between (C_7_H_6_N_3_O_2_)^+^ cations and tetrachlorozincate (II) entities. These entities are linked together through four types of H-bonds (Table S2 and Figure 2).

According to Figure 2, Zn (II) ions are surrounded by four chlorine atoms. Applying the Yang parameter τ_4_ [50], we can differentiate between the square plane geometry and the tetrahedral geometry. Thus, we specify the deformation rate of the geometries:

$$\tau_{4}=\frac{360-\left(\alpha +\beta \right)}{141}$$

For the [Zn(1)Cl_4_]^2−^ anions: α = 119.84 (5)°, β = 110.83 (5)° and τ_4_ = 0.917 and for [Zn(2)Cl_4_]^2−^ anions: α = 116.23 (5)°, β = 114.25 (5)° and τ_4_ = 0.918. Based on the values of τ_4_, we can affirm that the tetrachloridozincate (II) anions are slightly deformed tetrahedral. The d_Zn(1)–Cl_ varies between 2.2488 (11) and 2.3220 (12) Å for [Zn(1)Cl_4_]^2−^ and the d_Zn(2)–Cl_ varies from 2.2489 (12) to 2.3162 (13) Å. On the other side, the Cl–Zn(1)–Cl angles vary from 104.42 (4) to 119.84 (5)° and the $\hat{ClZn\left(2\right)Cl}$ vary from 104.25 (5) to 116.23 (5)° (Table S3). These values are very close to those found in works based on tetrachlorozincate (II) anions [51,52,53,54,55,56]. 

**Figure 3:**
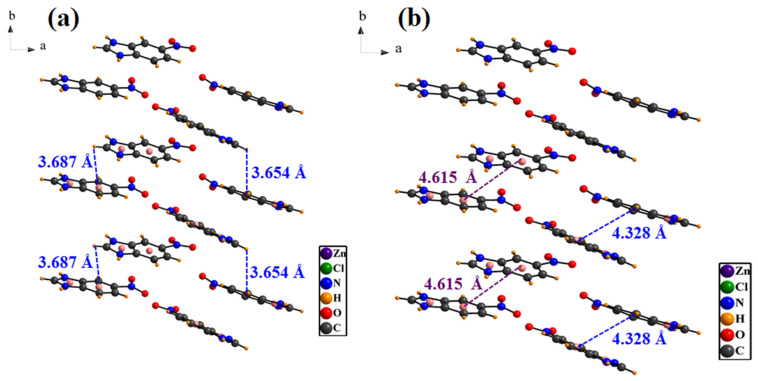
(**a**) C–H…π interactions; (**b**) π…π interactions.

In general, the cohesion of the crystal structure is ensured by different types of interactions. The main interaction in this structure is the hydrogen bonds which contribute to the stabilization of the crystal packing. Figure 2 states that the N–H and C–H moieties of the cation (C_7_H_6_N_3_O_2_)^+^ perform as H-bonds donors with the chlorine atoms of tetrachlorozincate (II) (C–H…Cl and N–H…Cl) and these fragments also act as donors of hydrogen bonds with the oxygen atoms of –NO_2_ group (C–H…O and N–H…O). The H-bond values vary from 3.1441 to 3.8471 Å (Table S2). As shown in Figure 3, the presence of C–H…π interactions in the crystal arrangement shows another type of interactions. The measurement of interaction values is determined by measuring the d (distance) between the centroid of the benzene values cycle and the C–H fragment of neighboring cations. This distance varies between 3.654 and 3.687 Å (Figure 3a) [57]. Figure 3b shows the weak π…π interactions (betwixt the centroids of two parallel aromatic rings).

The characteristic properties of the organic cations (C_7_H_6_N_3_O_2_)^+^ are displayed in Table S4. For the nitro groups, the d_N–O_ are located between 1.205 (5) and 1.223 (5) Å and the $\hat{ONO}$ angles vary from 118.2 (5) to 124.1 (6)°. For the benzimidazolium groups, the values of the d_N–C_ vary from 1.310 (5) to 1.475 (7) Å. The C–C distances vary between 1.356 (6) and 1.393 (5) Å. The values of$\hat{\;CCC}$, $\hat{NCC},$ and $\hat{NCN}\;$are betwixt 106.0 (4) and 133.0 (4)°. These values are almost homologous to the values obtained in 2-(3-hydroxypropyl) benzimidazoles [58]. 

### 3.2. HS Analysis, 2D Fingerprint Plots and E_XY_

**Figure 4:**
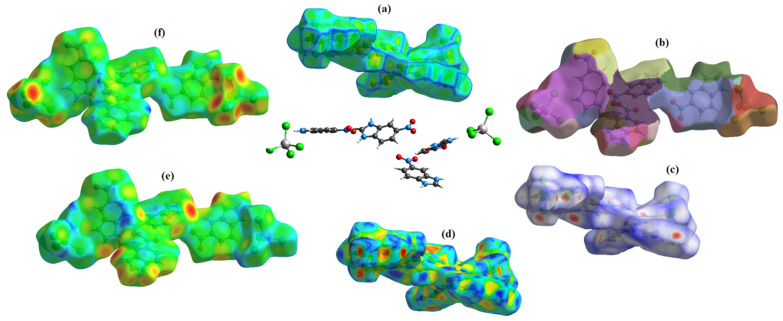
(**a**) Curvedness surface, (**b**) patch fragment surface, (**c**) d_norm_ surface, (**d**) shape index surface, (**e**) d_i_ and (**f**) d_e_ of (C_7_H_6_N_3_O_2_)_2_[ZnCl_4_].

**Figure 5:**
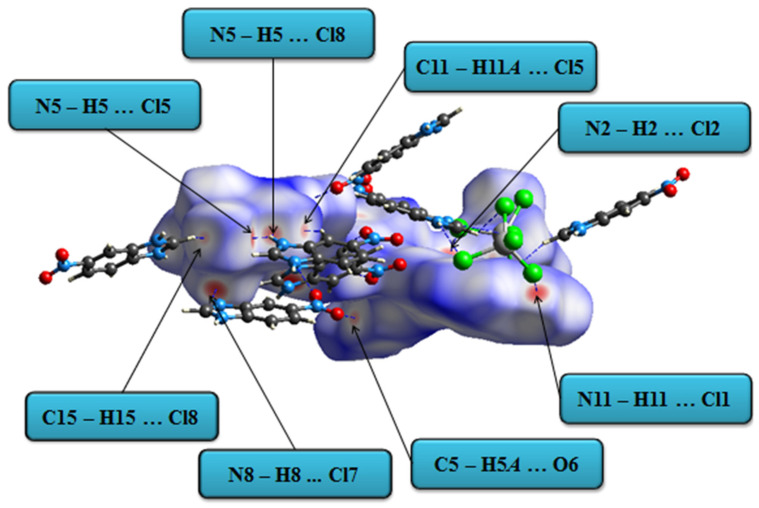
H-bonds present in (C_7_H_6_N_3_O_2_)_2_[ZnCl_4_] by d_norm_.

**Figure 6:**
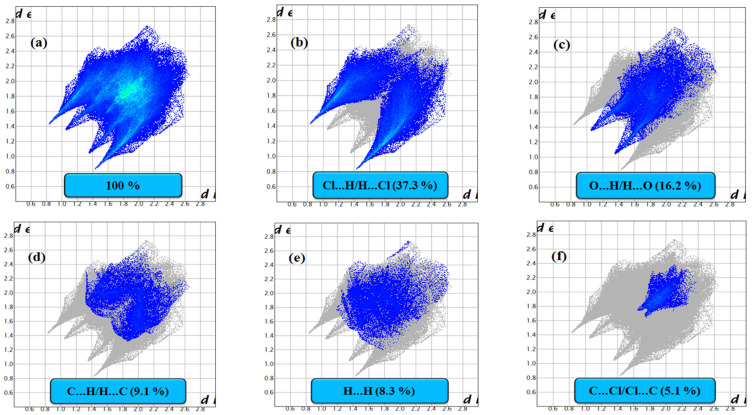
The 2D fingerprints plot of the intermolecular contacts presents in (C_7_H_6_N_3_O_2_)_2_[ZnCl_4_]: (**a**) 100%, (**b**) Cl…H/H…Cl contacts, (**c**) O…H/H…O contacts, (**d**) C…H/H…C contacts, (**e**) H…H contacts and (**f**) C…Cl/Cl…C contacts.

Exploiting CrystalExplorer 17.5 software [59], the Hirshfeld surfaces of (C_7_H_6_N_3_O_2_)_2_[ZnCl_4_] were mapped over curvedness (range from −4.0000 to 0.4000), patch fragment (vary between 0.0000 and 49.0000), d_norm_ (vary between −0.4160 and 1.3942), shape index (vary between −1.0000 and 1.0000), d_i_ (range from 0.8343 to 2.6553 Å), and d_e_ (range between 0.8341 and 2.7385 Å) (Figure 4a–f). The red spots in the surface mapped on d_norm_ reveal that close contact interactions are observable near the Cl, N, C, and O atoms involved in the H-bonds (Table S2 and Figure 5). The presence of C–H…π interactions is strongly affirmed by the shape-index mapping. The two-dimensional fingerprint plots allow us to discuss all intermolecular contacts present in the crystal structure of our sample (Figure 6). The E_XY_ and E_XX_ were realized out of the actual contacts between the different entities and equiprobable proportions calculated from the chemical surface content.

In the crystalline structure of our compound, there are in fact fourteen H-bonds (Table S2). The H…Cl/Cl…H contacts are the favored interactions with d_e_ + d_i_ ~ 2.2 Å, and their contributions is of the order 37.3% (Figure 6b) with E_H…Cl_ = 1.79 > 1. This dominance is related to the abundance of chlorine (%S_Cl_ = 25.3%) and hydrogen (%S_H_ = 41.4%) at the molecular surface (Table 1). The second place of the dominant interactions in the crystalline structure is reserved for the H…O/O…H contacts with a contribution equal to 16.2% due to the abundance of hydrogen and oxygen (%S_O_ = 14.7%) on the molecular surface. In the fingerprint diagram, the H…O/O…H contacts (corresponding to N–H…X and C–H…X H-bonds with X = O) are viewed like a pair of symmetrical spikes with d_i_ + d_e_ ~ 2.4 Å) (Figure 6c). The percentage 9.1% is attributed to the C…H/H…C interactions representing the third most important interaction on the surface with an enrichment ratio below 1, E_C…H_ = 0.829 (Figure 6d). The H…C/C…H interactions correspond to the strong C–H…π interaction types. The H…H contacts are the fourth most frequent interactions materializing as a large region in the middle of the 2D fingerprint plot, forming 8.3% of the total Hirshfeld surface area (Figure 6e). Finally, 5.1% is attributed to C…Cl/Cl…C interactions. 

**Figure 7:**
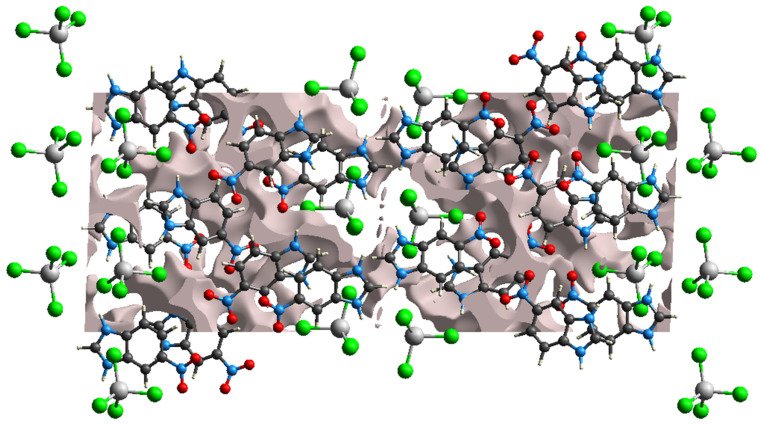
The projection along the *b*-axis showing the crystal voids in (C_7_H_6_N_3_O_2_)_2_[ZnCl_4_].

The CrystalExplorer software gives us the opportunity also to determine the void surface in the crystal structure of (C_7_H_6_N_3_O_2_)_2_[ZnCl_4_]. The calculation of the crystalline voids (0.002 a.u. isovalue) indicates that the void volume and the surface area of (C_7_H_6_N_3_O_2_)_2_[ZnCl_4_] are exactly equal to 381.08 Å^3^ and to 1285.09 Å^2^ respectively. The data given by the monocrystal XRD analysis showed that the volume of the unit cell is equal to 3995.8 Å^3^ (Table S1 and Figure 7). 9.54% represents the calculated porosity value and indicates that the cavities are not spacious. The electron density isosurfaces are not closed near the entities but are spaced out where there are H-bonds [60]. 

### 3.3. Infrared Spectroscopy

**Figure 8:**
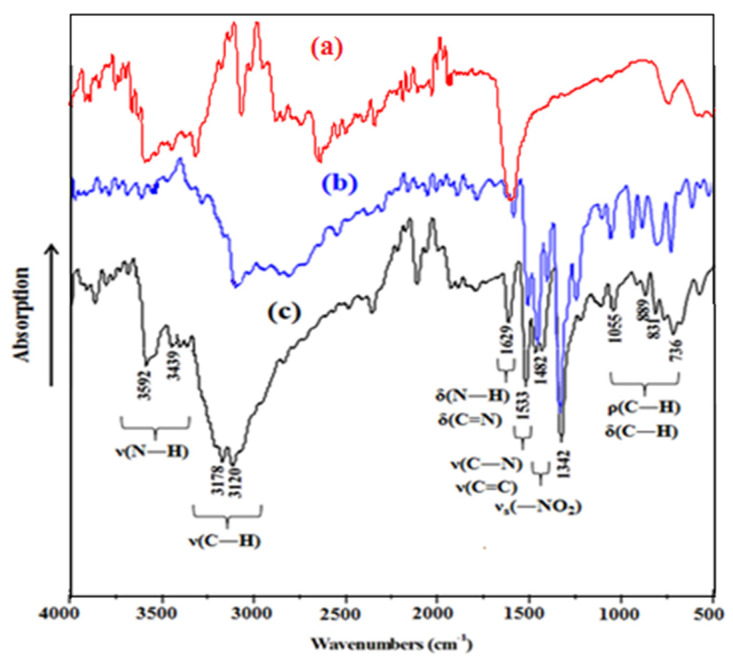
(**a**) IR spectra of ZnCl_2_; (**b**) IR spectra of 5-nitrobenzimidazole; (**c**) IR spectra of (C_7_H_6_N_3_O_2_)_2_[ZnCl_4_].

Spectral band assignments of the sample (C_7_H_6_N_3_O_2_)_2_[ZnCl_4_] are determined using IR spectra of similar compounds [61,62,63,64,65,66]. The experimental infrared spectrum in the range of 4000–500 cm^−1^ is shown in Figure 8. In this spectrum, specifically in the domain where the wavelength varies from 3600 to 3000 cm^−1^, the peaks detected at 3592 and 3439 cm^−1^ are reserved for N–H stretching vibrations, on the other hand, the bands sighted at 3178 and 3120 cm^−1^ are allotted to C–H vibration of the aromatic ring. The band around 1629 cm^−1^ is assigned to the stretching flexion of N–H and C=N. The band at 1533 cm^−1^ was attached to the C=C and C–N stretching vibrations. The organic cation (5-nitrobenzimidazolium) that participates in the synthesis of (C_7_H_6_N_3_O_2_)_2_[ZnCl_4_] has a nitro group which is bonded to the aromatic ring. In the previous research, asymmetric–symmetric stretching vibrations of –NO_2_ are normally observed between 1570 and 1485 cm^−1^ and between 1370 and 1320 cm^−1^ [67], respectively. The band spotted at 1482 cm^−1^ was reserved to ν_s_(–NO_2_) vibrations. The planar aromatic ρ(C–H) and δ(C–H) vibrations occur at 1055 and 736 cm^−1^, respectively.

### 3.4. Electric and Dielectric Reports 

#### 3.4.1. ε’ and ε″ versus Ln(f)

The dielectric characteristics of materials change relatively with the frequency of the applied electric field. Electrical permittivity is associated with dipole oscillations free in an alternating field which is demonstrated by Debye’s theory [68]. The complex permittivity ε* is bounded by the relation ε* = ε’(ω) − iε″(ω), so that ε’(ω) (the real part) exhibits the phenomenon of bound absorption to the energy storage capacity of the capacitor and ε″ (the imaginary part) shows the phenomenon of energy dissipation. They are calculated using the following equations:

$$\varepsilon^{\prime }\left(\omega \right)=\frac{1}{{\omega C}_{0}}\left[\frac{-Z\prime \prime }{{\;Z\varepsilon^{\prime }}^{2}+{\;Z\prime \prime }^{2}}\right];\;\varepsilon \prime \prime \left(\omega \right)=\frac{1}{{\omega C}_{0}}\left[\frac{Z\prime }{{\varepsilon^{\prime }}^{2}+{Z\prime \prime }^{2}}\right]\mathrm{with}\;C_{0}=\frac{\varepsilon_{0}\;S}{e}$$

ε_0:_ represent the geometric capacitance without dielectric (vacuum or air).

S: is the surface of the pellet which has the cylindrical shape.

e: called the thickness of the pellet.

**Figure 9:**
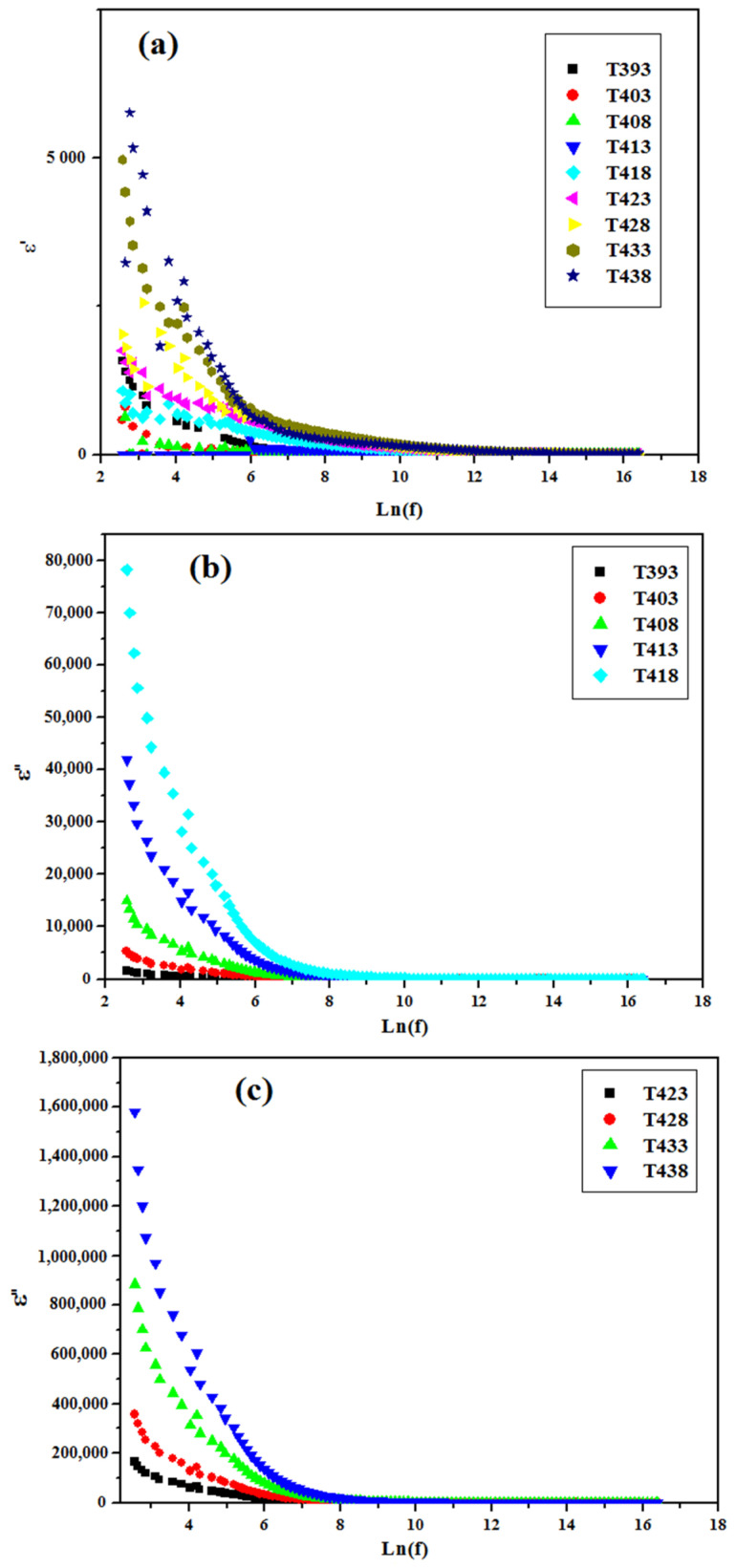
(**a**) ε’ versus Ln(f) in the temperature range from 393 to 438 K; (**b**) ε″ versus Ln(f) in the temperature range from 393 to 418 K; (**c**) ε″ versus Ln(f) in the temperature range from 423 to 438 K.

In Figure 9, we present the variation spectra of the real ε’ (a) and imaginary ε″ (b) part of the permittivity as a function of the frequency for a range of temperatures varying from 393 to 438 K. According to these graphs (a), (b), and (c), we notice that as the temperature increases, we see a strong increase in the permittivity at low frequency, followed by a decrease at high frequency in the vicinity of 10 Hz. The increase in the measurement temperature indicates an increase in the permittivity of the sample for ε″(b) and ε″(c); this is relatively defined by the significant presence of the relaxation time distribution in (C_7_H_6_N_3_O_2_)_2_[ZnCl_4_]. 

#### 3.4.2. Impedance Spectroscopy

**Figure 10:**
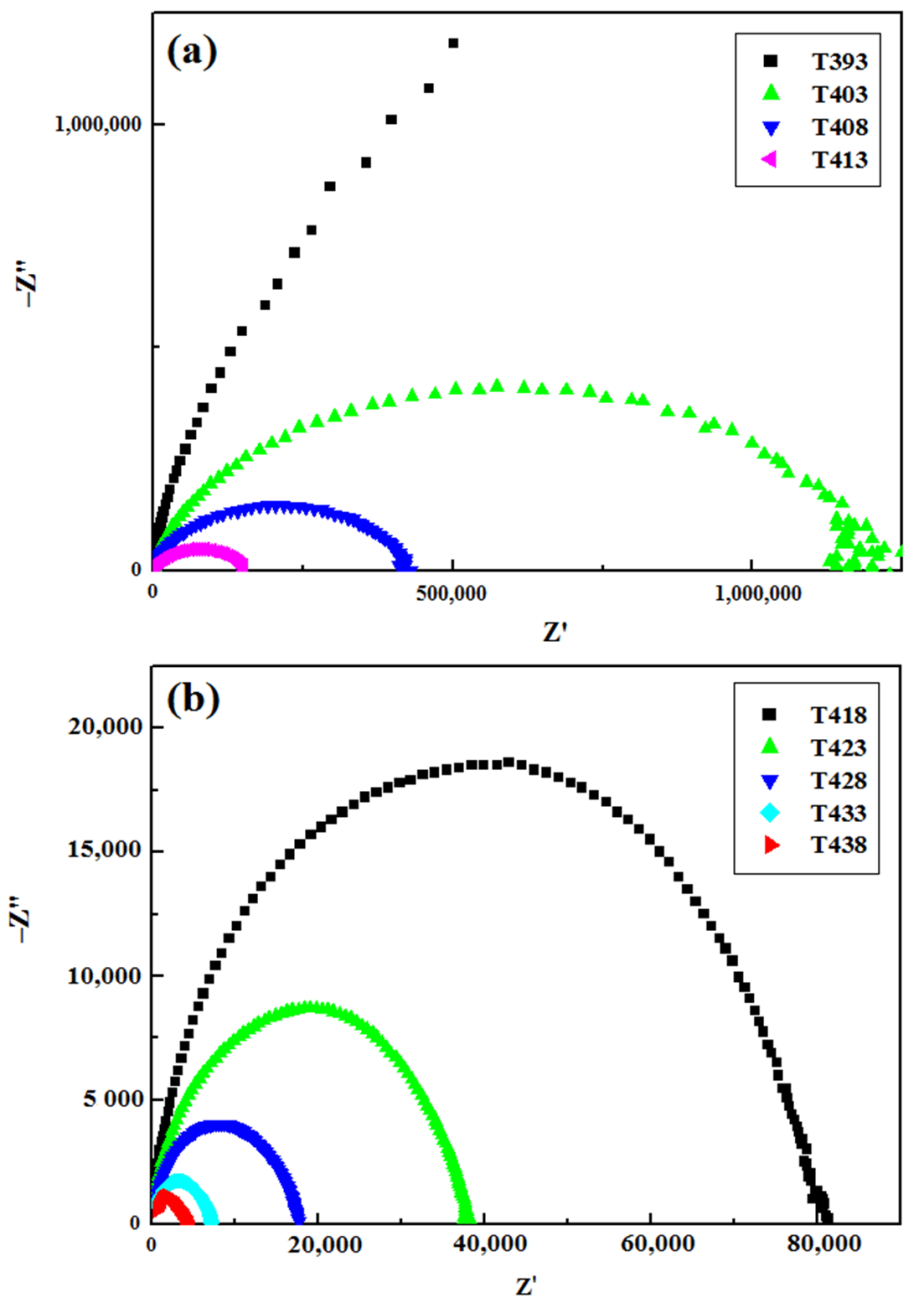
−Z″ versus Z’: (**a**) in the range 393–413 K and (**b**) in the range 418–438.

The Nyquist diagram −Z″ (imaginary part) versus (real part) of the studied compound at different temperatures (393 ≤ T ≤ 438 K) is shown in Figure 10. Well-defined semicircles passing through or near the origin were obtained for temperatures varying from 393 to 438 K. According to the diagrams announced in graphs (a) and (b), we can see that when the temperature increases, these circles get smaller and smaller, leading to an activated thermal conduction mechanism. 

**Figure 11:**
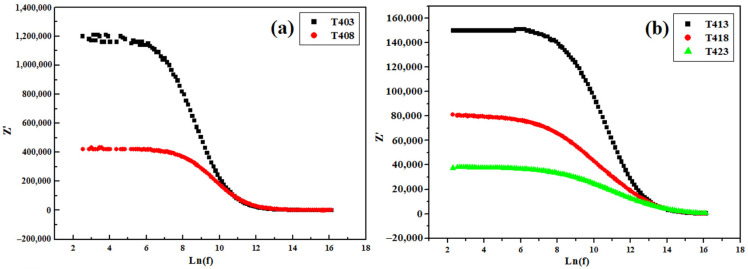

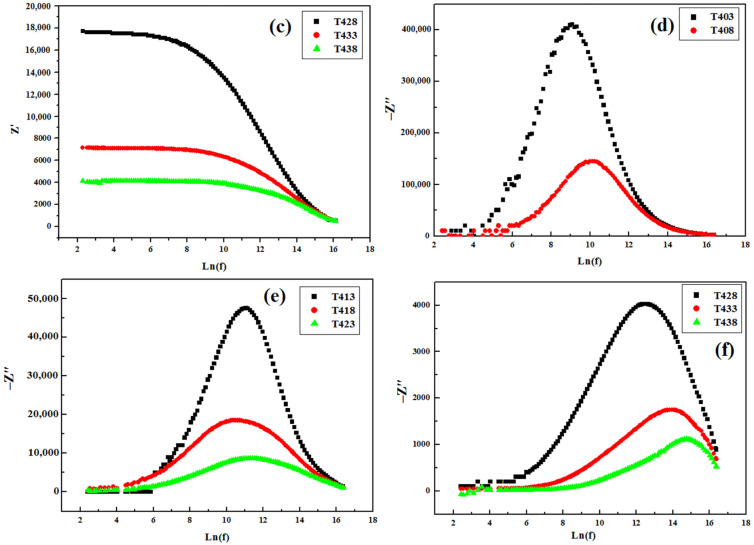
(**a**) Z’ versus Ln(f) in the range 403-408 K; (**b**) Z’ versus Ln(f) in the range 413-423 K; (**c**) Z’ versus Ln(f) in the range 428-438 K; (**d**) −Z″ versus Ln(f) in the range 403-408 K; (**e**) −Z″ versus Ln(f) in the range 413-423 K; (**f**) −Z″ versus Ln(f) in the range 428-438 K.

In Figure 11a–f, we show Z’ (real part) and Z″ (imaginary part) of the impedance versus Ln(f) at 393 ≤ T ≤ 438 K. The amplitude of Z’ decreases when the temperature and the frequency increase and consequently the AC conductivity increases. The phenomenon stated that the crystal comports as a semiconductor crystal. Z″ increases with the frequency until reaching a maximum peak (Z″ max), then we noticed a relaxation with the increase in the frequency. Moreover, the decrease in Z″ max values is related to the increase in temperature and localization toward the side of the high frequencies showing a single time of relaxation [69,70].

#### 3.4.3. Electric Conductivity

**Figure 12:**
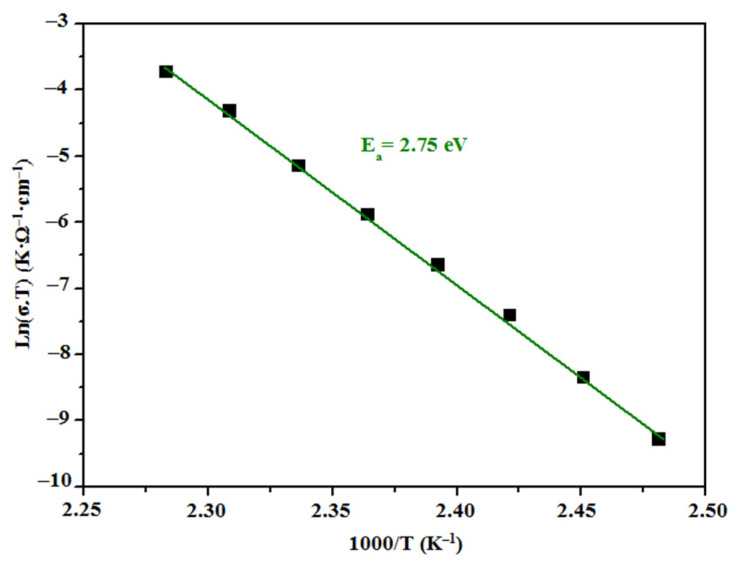
Ln(σ·T) versus $\frac{1000}{T}$.

Figure 12 shows an affine line explaining the variation of Ln(σ.T) versus 1000/T. This graph is explained by the formula of Arrhenius [71]:

σ·T = A exp (−E_a_/K_β_·T)

where E_a_ is the activation energy; A is the pre-exponential factor; K_β_ is the Boltzmann constant; T is the temperature in K. Using this straight line, we succeeded in determining the value of the activation energy such that its value is equal to E_a_ = 2.75 eV in the range of temperatures varying from 393 to 438 K. This value asserts that the transport mechanism is due to the thermally activated hopping process.

#### 3.4.4. Electrical Modulus 

The electrical characteristics of the compound (C_7_H_6_N_3_O_2_)_2_[ZnCl_4_] can be evaluated by applying complex electrical modulus formalism. This alternative approach is equally adequate to identify observable phenomenon in the compound such as the polarization of the electrodes and the relaxation times of the conductivity [72]. The complex electrical modulus can be stated by the following formula:

$$M*\;(\omega )=\frac{1}{\varepsilon^{*}}=M^{\prime }+\mathrm{jM}\prime \prime$$

where M’ = ΩC_0_Z″, M″ = ΩC_0_Z’, ω represents the angular frequency (2πf), and C_0_ = ε_0_(A/t) is the geometrical capacitance.

**Figure 13:**
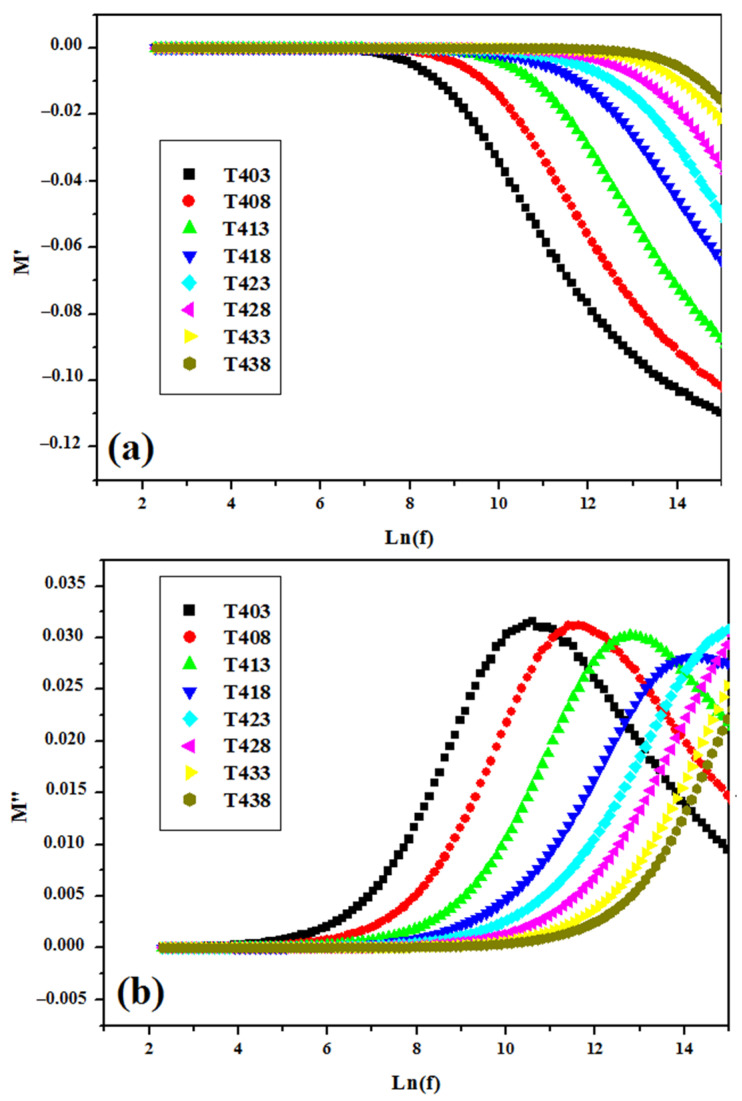
(**a**) M’ versus Ln(f) in the range 403–438 K and (**b**) M″ versus Ln(f) in the range 403–438 K.

The graphs in Figure 13 present M’ and M″ (real and imaginary parts respectively) of the electrical modulus M versus Ln(f) in temperature interval of 393–438 K. In the graph (a), M’ values are low in the low frequency region and gradually increase with frequency. On the other side in the graph, (b) M″ declares a maximum at a frequency ω_max_ highlighting the relaxation phenomenon of the system. Thus, we can notice that the position of the peak M″ max approaches toward higher frequencies as the temperature increases. Therefore, the presence of such relaxation peaks in the plot M″ shows that one can consider the samples as ionic conductors [73]. Accordingly, the frequency region below the M″ max peak assigns the range in which H^+^ charge carriers are mobile over a long distance. Nevertheless, the frequency regions above the M″ max peak designate the range in which carriers are confined to potential wells and are mobile over short distances.

### 3.5. Molecular Docking Details

**Figure 14:**
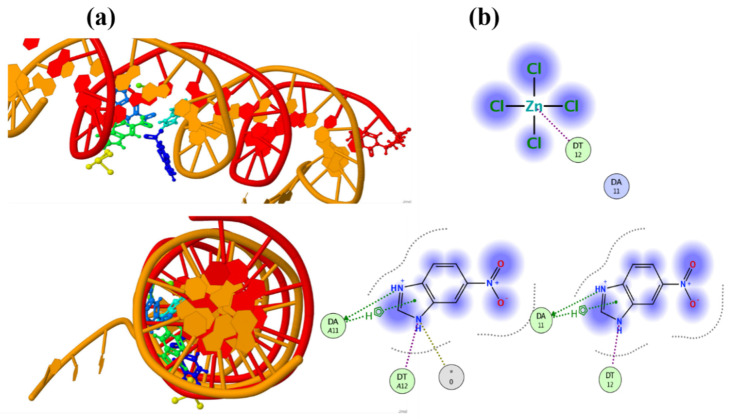
Side and lop view of molecular docked complex (C_7_H_6_N_3_O_2_)_2_[ZnCl_4_] (**a**) and its ligplots (**b**) determined by PM3 semi-empirical level of theory.

The molecular mechanism and modes of interactions in the title compound with double-stranded DNA were interpreted by means of molecular docking approach. The reasoning of the pose view and conformations of the compound with the lowest free energy are shown in Figure 14. Binding free energy (Δ*G*) and binding constants “*Kb*” value of (C_7_H_6_N_3_O_2_)_2_[ZnCl_4_] compound are reported in Table 2. 

Figure 14a side view and top view represented the best possible interactions of the title material with DNA through groove binding with the grooves of DNA. A disordered structure led to different interactions with different parts. It is evident from the ligplot, Figure 14b that here ZnCl_2_ interacted via electrostatic with negatively charged thymine DT(12) and aromatic fragments 2D ligplot exhibited arene–arene interaction with adenine DA (11) and donor-acceptor interactions with thymine DT (12) also represented as DT (A12). Binding constant ″*Kb*″ and free energy ΔG indicated that the interaction of (C_7_H_6_N_3_O_2_)_2_[ZnCl_4_] compound with DNA is spontaneous with sufficient binding propensity [74,75].

For the comprehensive understanding of physicochemical interactions of the complex, a number of electronic and steric descriptors are determined and are grouped in Table 3 and Table 4. E_HUMO_ and E_LUMO_ values provided an estimate of the electron-donating or electron-accepting character of a given compound and, therefore, a compound is believed to be more electron-donating as the value of its E_HOMO_ escalates and more electrons accepting as the value of its E_LUMO_ declines [76]. Results depicted that our compound acts as an electron acceptor during its interactions with DNA base pairs. While interacting with DNA, (C_7_H_6_N_3_O_2_)_2_[ZnCl_4_] which draws electrons from electron-rich base pairs and performs as a good electron acceptor defines the reason for higher binding value of the title material with DNA. On the other hand, steric descriptors also displayed a reasonably good correlation with binding constant, “*K_b_*”. SlogP termed as partition coefficient determined the extent of lipophilicity of compounds indicating the complex is lipophilic in nature. Molar refractivity (MR) is another important steric descriptor which measures polarizability of the molecule [77]. The compounds having greater polarizability have greater tendency of its electronic cloud distortion and overlap with DNA base pairs, hence (C_7_H_6_N_3_O_2_)_2_[ZnCl_4_] exhibited greater potential of interactions with DNA base pairs (Table 4).

In conclusion, molecular docking is a powerful technique to find binding of compounds with biological macromolecule and allows the identification of correct intermolecular binding conformation. The compound (C_7_H_6_N_3_O_2_)_2_[ZnCl_4_] revealed good binding energy with all targets of the compound which showed an excellent binding energy established on the interaction between cationic and anionic entities [78,79].

## 4. Conclusions

Summarizing, the compound (C_7_H_6_N_3_O_2_)_2_[ZnCl_4_] crystallized in monoclinic system *P*2_1_/*c* as evidenced by the results obtained by X-ray diffraction. The atomic arrangement can be described by alternation between cations and anions in the (ac) plane. The crystal structure of bis(5-nitrobenzimidazolium) tetrachlorozincate (II) is stabilized by four types of H-bonds and different types of interactions forming a 3D architecture. The HS allowed us to show that the H…Cl/Cl…H (37.3%) contacts are the most frequent in the crystalline structure. IR spectroscopy was manipulated to substantiate the presence of different functional groups in the compound. Temperature and frequency have important roles in showing dielectric characteristics in the range 393–438 K. Arrhenius’ law governs relaxation time and electrical conductivity. The conductivity of this material was examined as a function of frequency in the temperature range 393–438 K, where the conduction process attributed to the ion-hopping mechanism. Finally, the docking study revealed that compound interacted with DNA efficiently with significant binding strength exhibiting antibacterial activity.

## Figures and Tables

**Table 1:** Enrichment reports (E_XY_) of (C_7_H_6_N_3_O_2_)_2_[ZnCl_4_].

Atoms	Zn	Cl	N	O	C	H
**% Surface**	**0.8**	**25.3**	**4.85**	**14.7**	**13.35**	**41.1**
**Zn**	**-**	0.988			**-**	0.760
**Cl**		0.249	1.344			
**N**				0.771	**-**	0.627
**O**		0.174		1.52	1.070	1.340
**C**		0.754			1.402	0.829
**H**		1.793				0.491

**Table 2:** “Kb” and Δ*G* values for the ds DNA complexes calculated from molecular docking data.

Complex Code	Molecular Docking	
	″*Kb*″/M^−1^	(−Δ*G*) KJ·mol^−1^
(C_7_H_6_N_3_O_2_)_2_[ZnCl_4_]	7.99 × 10^7^	45.01

**Table 3:** Details of electronic descriptions calculated from molecular docking data.

Complex	E_HOMO_	E_LUMO_	E_ele_	E_IP_	E_Total_
(C_7_H_6_N_3_O_2_)_2_[ZnCl_4_]-DNA	−16.88	−9.36	−2,294,329.0	16.88	−275,981.313

**Table 4:** Details of steric descriptors calculated from molecular docking information.

Complex	M_R_	H_f_	*SlogP*	Dipole
(C_7_H_6_N_3_O_2_)_2_[ZnCl_4_]-DNA	24.61	887.22	7.61	33.59

## Data Availability

This statement is excluded.

## Supplementary Materials

The following supporting information can be downloaded at: https://www.mdpi.com/article/10.3390/ma15227973/s1, Table S1: The crystallographic details of (C_7_H_6_N_3_O_2_)_2_[ZnCl_4_]; Table S2: The different H-bonds present in (C_7_H_6_N_3_O_2_)_2_[ZnCl_4_]; Table S3: The different values of d_Zn–Cl_ and $\hat{\mathrm{ClZnCl}}$ in [ZnCl_4_]^2−^ anions; Table S4: The different characteristics of (C_7_H_6_N_3_O_2_)^+^ cation.
