# Supplementary material for: A New Zn(II) Metal Hybrid Material of 5-Nitrobenzimidazolium Organic Cation (C7H6N3O2)2[ZnCl4]: Elaboration, Structure, Hirshfeld Surface, Spectroscopic, Molecular Docking Analysis, Electric and Dielectric Properties
Source: Materials (Basel). 2022 Nov 11;15(22):7973. doi: 10.3390/ma15227973 (PMC9697581; doi:10.3390/ma15227973)
Supplement: Supplementary file 1 [file materials-15-07973-s001.zip › materials-1845242-supplementary.pdf]

Supplementary Information

A New Zn(II)Metal HybridMaterial of 5-Nitrobenzimidazolium Organic Cation $(C_7H_6N_3O_2)_2[ZnCl_4]$: Elaboration, Structure, Hirshfeld Surface, Spectroscopic, Molecular Docking Analysis, Electric and Dielectric Properties

Chaima Ayari [1], Abdullah A. Alotaibi [2,*], Mohammed A. Baashen [2], Fouzia Perveen [3], Abdulhadi H. Almarri [4], Khalid M. Alotaibi [5], Mohammed S. M. Abdelbaky [6], Santiago Garcia-Granda [6], Abdelhak Othmani [7], Cherif Ben Nasr [1] and Mohamed Habib Mrad [1,2]

[1] Materials Chemistry Laboratory, Faculty of Sciences of Bizerte, University of Carthage, Zarzouna, Bizerte 7021, Tunisia
[2] Department of Chemistry, College of Sciences and Humanities, Shaqra University, Ad-Dawadmi 11911, Saudi Arabia
[3] School of Interdisciplinary Engineering and Sciences (SINES), NUST, H-12, Islamabad 44000, Pakistan
[4] Department of Chemistry, University College of Al-Wajah, University of Tabuk, Tabuk 71421, Saudi Arabia
[5] Department of Chemistry, College of Science, King Saud University, Riyadh 12271, Saudi Arabia
[6] Department of Physical and Analytical Chemistry, University of Oviedo-CINN, 33006 Oviedo, Spain
[7] Laboratory of Material Physics: Structures and Properties, LR01 ES15, Faculty of Sciences, University of Carthage, Zarzouna, Bizerte 7021, Tunisia
* Correspondence: aaalotaibi@su.edu.sa

Table S1. The crystallographic details of $(C_7H_6N_3O_2)_2[ZnCl_4]$.

Crystal Data	Results
Chemical formula	$(C_7H_6N_3O_2)_2[ZnCl_4]$
Molar mass ($g.mol^{-1}$)	535.49
Crystal system, Space group	Monoclinic, $P2_1/c$
T (K)	293
Cell parameters	a = 36.7954 (13), b = 7.2299 (2) and c = 15.0307 Å<br>β = 92.118°<br>V = 3995.8
Z	8
Radiation type	Mo $K\alpha$ (λ= 0.71073 Å)
μ (mm^{-1})	1.80
Crystal dimensions (mm)	0.28×0.16×0.07
**Data Collection**	
Diffractometer	Xcalibur, Ruby, Gemini
Absorption correction	Multi-scan
θ_{min}, θ_{max} (°)	2.7, 31.5
T_{min}, T_{max}	0.715, 0.882
Limits h, k, l	h = –53→54<br>k = –10→10<br>l = –21→21
No. of measured, independent and observed [$I>2\sigma(I)$] reflections	12402, 12402, 5614
R_{int}	0.115
$(\sin\theta/\lambda)_{max}$ ($Å^{-1}$)	0.734
**Refinement**	
$R [F^2>2\sigma(F^2)], wR (F^2), S$	0.063, 0.114, 1.03
No. of reflections	12402
No. of parameters	523
$\Delta\rho_{min}$, $\Delta\rho_{max}$ (e.$Å^{-3}$)	–0.57, 0.50
**CCDC No.**	**2166515**

Table S2. The different H-bonds present in $(C_7H_6N_3O_2)_2[ZnCl_4]$.

D*–H … *A	***D*–H (Å)**	**H … *A* (Å)**	***D* … *A* (Å)**	***D*–H … *A* (°)**
**N1 – H1 … Cl2**	0.86	2.36	3.1441	152
**N2 – H2 … Cl2**	0.86	2.65	3.2901	132
**N2 – H2 … O8**	0.86	2.35	3.8471	138
**N4 – H4 … Cl5**	0.86	2.48	3.2009	156
**N5 – H5 … Cl5**	0.86	2.67	3.3480	137
**N5 – H5 … Cl8**	0.86	2.62	3.2599	132
**N7 – H7 … Cl6**	0.86	2.44	3.1745	144
**N8 – H8 … Cl7**	0.86	2.52	3.2521	143
**N10 – H10 … Cl1**	0.86	2.39	3.1454	147
**N11 – H11 … Cl1**	0.86	2.55	3.2033	133
**C1 – H1*A* … Cl3**	0.93	2.80	3.3778	122
**C5 – H5*A* … O6**	0.93	2.51	3.2293	135
**C8 – H8*A* … Cl7**	0.93	2.64	3.4471	146
**C11 – H11*A* … Cl5**	0.93	2.76	3.4721	134
**C13 – H13 … O7**	0.93	2.54	3.669	148
**C15 – H15 … Cl8**	0.93	277	3.4023	126
**C15 – H15 … Cl6**	0.93	2.82	3.3823	120

Table S3. The different values of d_{Zn-Cl} and $\widehat{ClZnCl}$ in $[ZnCl_4]^{2-}$ anions.

**Distances (Å)**		**Angles (°)**	
**$[Zn(1)Cl_4]^{2-}$**			
Zn1 – Cl1	2.2655 (12)	Cl1 – Zn1 – Cl2	107.18 (5)
Zn1 – Cl2	2.3220 (12)	Cl4 – Zn1 – Cl2	104.42 (4)
Zn1 – Cl3	2.2488 (11)	Cl3 – Zn1 – Cl2	105.07 (5)
Zn1 – Cl4	2.2596 (11)	Cl4 – Zn1 – Cl1	110.83 (5)
		Cl3 – Zn1 – Cl1	108.60 (4)
		Cl3 – Zn1 – Cl4	119.84 (5)
**$[Zn(2)Cl_4]^{2-}$**			
Zn2 – Cl5	2.3162 (13)	Cl6 – Zn2 – Cl8	116.23 (5)
Zn2 – Cl6	2.2489 (12)	Cl6 – Zn2 – Cl7	107.51 (4)
Zn2 – Cl7	2.2610 (12)	Cl8 – Zn2 – Cl7	114.25 (5)
Zn2 – Cl8	2.2581 (13)	Cl6 – Zn2 – Cl5	108.35 (5)
		Cl8 – Zn2 – Cl5	104.25 (5)
		Cl7 – Zn2 – Cl5	105.68 (5)

Table S4. The different characteristics of $(C_7H_6N_3O_2)^+$ cation.

**Distances (Å)**		**Angles (°)**	
**$(C_7H_6N_3O_2)^+$**			
N3 – O1	1.205 (5)	C15 – N7 – C16	109.1 (3)
N3 – O2	1.223 (5)	N8 – C15 – N7	109.7 (4)
N6 – O3	1.221 (6)	C18 – C17 – N8	131.7 (4)
N6 – O4	1.218 (6)	C18 – C17 – C16	122.1 (4)
N9 – O5	1.213 (5)	N8 – C17 – C16	106.2 (4)
N9 – O6	1.216 (5)	O1 – N3 – O2	125.4 (5)
N12 – O7	1.216 (5)	O1 – N3 – C6	118.2 (5)
N1 – C1	1.317 (5)	O2 – N3 – C6	116.4 (5)
N1 – C2	1.382 (5)	C27 – C28 – C23	115.3 (4)
N2 – C1	1.318 (5)	C19 – C18 – C17	115.0 (4)
N2 – C3	1.381 (5)	C1 – N1 – C2	109.2 (4)
N4 – C8	1.316 (6)	C22 – N11 – C24	109.3 (4)
N4 – C9	1.385 (6)	C15 – N8 – C17	109.0 (4)
N5 – C8	1.312 (6)	C22 – N10 – C23	109.8 (4)
N5 – C10	1.383 (6)	C1 – N2 – C3	109.7 (4)
N6 – C12	1.475 (7)	C8 – N5 – C10	108.7 (4)
N7 – C15	1.325 (5)	O5 – N9 – O6	123.2 (5)
N7 – C16	1.380 (5)	O5 – N9 – C19	118.5 (4)
N8 – C15	1.317 (5)	O6 – N9 – C19	118.2 (4)
N8 – C17	1.383 (5)	N11 – C24 – C25	132.7 (4)
N9 – C19	1.470 (6)	N11 – C24 – C23	106.0 (4)
N10 – C22	1.310 (5)	C25 – C24 – C23	121.3 (4)
N10 – C23	1.371 (5)	N7 – C16 – C21	132.7 (4)
N11 – C22	1.324 (5)	N7 – C16 – C17	106.0 (4)
N11 – C24	1.371 (5)	C21 – C16 – C17	121.3 (4)
N12 – C27	1.481 (6)	O7 – N12 – O8	123.9 (5)
C2 – C3	1.385 (5)	O7 – N12 – C27	117.8 (5)
C2 – C7	1.379 (6)	O8 – N12 – C27	118.4 (5)
C3 – C4	1.386 (6)	C28 – C27 – C26	124.0 (5)
C4 – C5	1.377 (6)	C28 – C27 – N12	117.4 (4)
C6 – C5	1.390 (6)	C26 – C27 – N12	118.6 (5)
C7 – C6	1.363 (6)	C18 – C19 – C20	125.1 (4)
C10 – C9	1.385 (6)	C18 – C19 – N9	118.1 (4)
C10 – C11	1.371 (6)	C20 – C19 – N9	116.8 (4)
C11 – C12	1.356 (6)	C7 – C2 – N1	131.2 (4)
C9 – C14	1.387 (7)	C7 – C2 – C3	122.5 (4)
C12 – C13	1.388 (7)	N1 – C2 – C3	106.4 (4)
C13 – C14	1.369 (7)	N10 – C23 – C28	131.8 (4)
C16 – C17	1.394 (5)	N10 – C23 – C24	105.9 (4)
C17 – C18	1.377 (6)	C28 – C23 – C24	122.2 (4)
C19 – C18	1.364 (6)	C26 – C25 – C24	117.1 (4)
C19 – C20	1.393 (5)	N10 – C22 – N11	109.0 (4)
C20 – C21	1.376 (6)	N2 – C3 – C2	105.6 (4)

C16 – C21	1.391 (6)	N2 – C3 – C4	133.0 (4)
C24 – C23	1.391 (5)	C2 – C3 – C4	121.3 (4)
C24 – C25	1.388 (6)	C11 – C10 – N5	131.8 (4)
C23 – C28	1.373 (6)	C11 – C10 – C9	121.5 (5)
C25 – C26	1.368 (6)	N5 – C10 – C9	106.7 (4)
C27 – C26	1.390 (6)	C8 – N4 – C9	109.4 (4)
C27 – C28	1.368 (6)	C6 – C7 – C2	115.1 (4)
		C25 – C26 – C27	120.1 (4)
		C12 – C11 – C10	115.9 (5)
		C7 – C6 – C5	124.0 (5)
		C7 – C6 – N3	118.7 (5)
		C5 – C6 – N3	117.4 (5)
		C21 – C20 – C19	119.1 (4)
		C20 – C21 – C16	117.4 (5)
		O4 – N6 – O3	124.1 (6)
		O4 – N6 – C12	118.2 (6)
		O3 – N6 – C12	117.8 (5)
		N1 – C1 – N2	109.0 (4)
		C10 – C9 – N4	105.4 (5)
		C10 – C9 – C14	121.5 (5)
		N4 – C9 – C14	133.1 (5)
		C11 – C12 – C13	124.3 (5)
		C11 – C12 – N6	118.1 (5)
		C13 – C12 – N6	117.6 (5)
		C5 – C4 – C3	116.8 (4)
		N5 – C8 – N4	109.8 (5)
		C4 – C5 – C6	120.2 (5)
		C14 – C13 – C12	119.4 (6)
		C13 – C14 – C9	117.3 (5)
